# Antinociceptive Effects of H_3_ (R-Methylhistamine) and GABA_B_ (Baclofen)-Receptor Ligands in an Orofacial Model of Pain in Rats

**DOI:** 10.1007/s12640-013-9385-4

**Published:** 2013-03-06

**Authors:** Przemysław Nowak, Magdalena Kowalińska-Kania, Damian Nowak, Richard M. Kostrzewa, Jolanta Malinowska-Borowska

**Affiliations:** 1 Department of Toxicology and Health Protection, Medical University of Silesia, Medyków 18 Str, 40-752 Katowice, Poland; 2 M&B Kania Dental Clinic, Panewnicka 168 Str, 40-772 Katowice, Poland; 3 Department of Toxicology and Health Protection (Student Circle), Medical University of Silesia, Medyków 18 Str, 40-752 Katowice, Poland; 4 Department of Biomedical Sciences, Quillen College of Medicine, East Tennessee State University, P.O. Box 70577, Johnson City, TN 37614 USA

**Keywords:** H_3_ receptor, GABA_B_ receptor, Orofacial pain, Formalin test, Rats

## Abstract

The present study explored the antinociceptive effects of H_3_ (R-alpha-methylhistamine) and GABA_B_ (baclofen) receptor ligands in an orofacial model of pain in rats. Orofacial pain was induced by subcutaneous injection of formalin (50 μl, 5 %) in the upper lip region, and the number of jumps and time spent face rubbing was recorded for 40 min. Formalin produced a marked biphasic pain response; first phase, 0–10 min (jumps), and second phase, 15–40 min, (rubbing). Baclofen (50 μg) injected into the rat wiskerpad 5 min before formalin administration suppressed both phases of pain whereas R-alpha-methylhistamine (12.5 μg) abolished the first phase only. Brains were taken immediately after behavioral testing was completed. HPLC/ED analysis showed that 5-hydroxytryptamine (5-HT) turnover was increased in hippocampus, thalamus, and brain stem of all formalin groups, excepting the baclofen group in which the balance of 5-HT metabolism was restored to control values. These findings demonstrate that GABA_B_ receptors represent peripheral targets for analgesia. Consequently, locally administered baclofen may be a useful approach in treating inflammatory trigeminal pain.

## Introduction

Pain is the primary reason for patients to visit a physician or dentist, and the diagnosis and management of pain in the face, mouth and jaws have been integral components of dental practice. However, for an adequate understanding of the neurological mechanisms underlying dental pain, with the objective of improving pain management, an animal model for dental pain assessment is frequently employed. Such models allow us to manipulate neural pathways for the study of those pathways and search for new treatment options (Raboisson and Dallel 2004; Rusina et al. 2010).

Among variety of different systems and associated receptors mediating nociception transmission and modulation, GABA_B_ and histamine H_3_ receptors have recently attracted most attention. GABA_B_ is the principle inhibitory metabotropic receptor in the central nervous system of mammals. Activation of the GABA_B_ receptor leads to the blockade of voltage-gated calcium channels, which results in inhibition of presynaptic mediator release as well as the inhibition of postsynaptic neuronal activity by indirect activation of K^+^ conductance (Koyrakh et al. 2005; Mapelli et al. 2009). Baclofen, a specific agonist of the heterodimeric GABA_B_ receptor, is effectively used in the treatment of rigidity and spasms of skeletal muscles. Furthermore, baclofen also induces antinociception in several animal pain models (Franek et al. 2004; Potes et al. 2006). Its antinociceptive mechanism involves inhibition of glutamate release from Adelta and C primary afferent terminals in substantia gelatinosa and/or a decreased release of neurokinin in the spinal cord (Ataka et al. 2000; Lao and Marvizón 2005).

In the past 10–15 years, the neuronal histaminergic system in brain has become reasonably well characterized (Pollard et al. 1993; Brown et al. 2001). Cell bodies of histaminergic neurons are located exclusively in the tuberomammillary nuclei of the hypothalamus and give rise to widespread projections throughout the central nervous system, including subcortical nuclei and cerebral cortex. Four subtypes (H_1_, H_2_, H_3_, H_4_) of histamine receptors are currently recognized. The histamine H_3_ subtype is the least defined, although histamine H_3_ receptors are now known to be predominately located presynaptically, functioning as an autoreceptor that regulates the synthesis and release of histamine (Flik et al. 2011). Recently, histaminergic agonists and antagonists were shown to modulate antinociception induced by supraspinally administered mu-, epsilon-, delta-, and kappa-opioid receptor agonists (Suh et al. 1999). Histaminergic agonists and antagonists additionally modulate peripheral opioid-mediated antinociception (Fernández-Dueñas et al. 2010a) and may be involved in the regulation of nociception during cholestasis in rats (Hasanein 2010).

To the best of our knowledge, there are no literature data on locally applied histamine H_3_ and GABA_B_ receptor agonists on trigeminal mediated nociception. In an attempt to clarify this issue, we produced a model orofacial formalin test in rats and associated effects with monoamine levels in brain.

## Materials and Methods

### Animals and Treatment

Adult (8–10 weeks of age) male and female Wistar rats weighting 200–250 g were obtained from the University Animals Department (Katowice, Poland) and were housed in a well-ventilated room, at 22 ± 2 °C under a 12 h light:12 h dark cycle (lights on 7:00 a.m. to 7:00 p.m.), and with free access to food and water. All procedures were approved by the Local Bioethical Committee for Animal Care (permission no 62/2011 issued on 2011.09.14) and are in accord with principles and guideline described in the NIH booklet Care and Use of Laboratory Animals. Experiments were carried out in the morning and the animals were used only once.

### Orofacial Formalin Test

Assessment of pain transmitted by trigeminal sensory pathway was evaluated by the orofacial formalin test in the rats (Park et al. 2011). Rats were divided into 4 groups (8 rats in each group) and placed in a clear plastic test chamber (30 × 30 × 30 cm) with three mirrored sides. Following a 30 min acclimation period, saline (50 μl) (2 groups), R- alpha-methylhistamine (12.5 μg/50 μl; 1 group), or baclofen (50 μg/50 μl; 1 group) was injected subcutaneously (30-gauge needle) into the right upper lip. Immediately afterward, rats were returned to the test chamber for 5 min, then injected again in the right upper lip with saline (50 μl) (1 control group) or 5 % formalin solution (remaining groups). Rats were then returned to the chambers, and nociceptive behavior was observed. Two parameter were evaluated, numbers of jumps (observed mainly in the first 10 min of testing) and time that animals spent rubbing and flicking (15–40 min). A reduction of formalin-induced behavior observed after administration of a given drug is interpreted as an antinociceptive response.

### Assessment of biogenic amine and metabolite content

Immediately after behavioral testing rats were decapitated, and the frontal cortex, thalamus, and spinal cord were rapidly dissected and placed on dry ice, then weighed and stored at –70 °C, pending assay. Samples were homogenized for 15–20 s in ice-cold trichloracetic acid (0.1 M), containing 0.05 mM ascorbic acid. After centrifugation (5,000×g, 5 min), supernatants were filtered through 0.2 μm cellulose membranes (Titan MSF Microspin filters, Scientific Resources Inc., Eatontown GB) and supernatants was injected onto the HPLC/ED column. Levels of noradrenaline (NA), dopamine (DA), 3,4-dihydroxyphenylacetic acid (DOPAC), 5-hydroxytryptamine (5-HT) and 5-hydroxyindoleacetic acid (5-HIAA) were assayed. The composition of the mobile phase was: 75 mm NaH_2_PO_4_, 1.7 mm 1-octanesulfonic acid, 5 μm EDTA (Avocado, Research Chemical Ltd., Morecambe, GB), 100 μl triethylamine (Sigma, St. Louis, USA), 9.5 % acetonitrile (J.T. Baker, Deventer, Holland), pH 3 adjusted with phosphoric acid (Fluka, Steinheim, Switzerland). The flow rate was maintained at 0.7 ml/min, at a temperature of 22 °C, and the oxidation potential was fixed at +700 mV, 10 nA/V sensitivity. Peaks were automatically integrated by universal chromatographic interface UCI-100 (Dionex Softron Gmbh, Germering, Germany). The instrumentation included an electrochemical detector (Gilson, Villiers-le-Bel, France) model 141 with flow cell, piston pump model 302 with head 5SC (Gilson, Villiers-le-Bel, France), manometric module model 802 (Gilson, Villiers-le-Bel, France), thermostat for STH 595 column (Dionex Softron Gmbh, Germering, Germany), precolumn Hypersil BDS C18, 10 × 4 mm, 3 μm (ThermoQuest, Waltham, GB) and chromatographic column Hypersil BDS C18, 250 × 4.6 mm, 3 μm (ThermoQuest, Waltham, GB). The data were quantified using the area under the peaks and external standards, using Chromeleon software (Dionex, Germany) (Nowak et al. 2006; Korossy-Mruk et al. 2013).

### Data Analysis

Group differences were assessed by an analysis of variance (ANOVA) and the post-ANOVA test of Newman–Keuls. A *P* value <0.05 was taken as the level of significant difference.

## Results

### Orofacial Formalin Test

Injection of formalin in the rat wiskerpad induces behavioral responses like jumping and rubbing. It consists of two distinct phases: a first “phasic” phase and a second “tonic” phase. In phase I called early or neurogenic (0–10 min) rats mainly jump; in phase II (late or inflammatory) (15–40 min) rats mainly rub their snout.

**Fig. 1:**
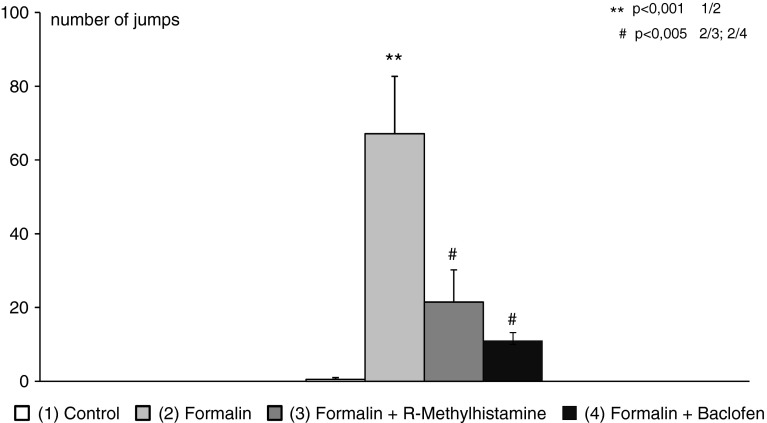
Anti-nociception effects measured by numbers of jumps after locally applied R-methylhistamine (12.5 μg) and baclofen (50 μg), assessed in the orofacial formalin test in rats (x ± SEM; *n* = 8). *Open square* Control, *Light grey box* Formalin, *dark grey box* Formalin + R-alpha-methylhistamine, *black box* Formalin + baclofen

In the formalin group, we observed ~67.0 (±15.7) jumps during time testing, control animals (saline + saline) did not express this behavior at all. R-alpha-methylhistamine (12.5 μg) injected before formalin administration significantly diminished numbers of jumps (to 21.5 ± 8.76). Also, baclofen in a dose of 50 μg applied locally in the rat wiskerpad 5 min before formalin injection, reduced jumping behavior (to 11.0 ± 2.19) (Fig. 1).

**Fig. 2:**
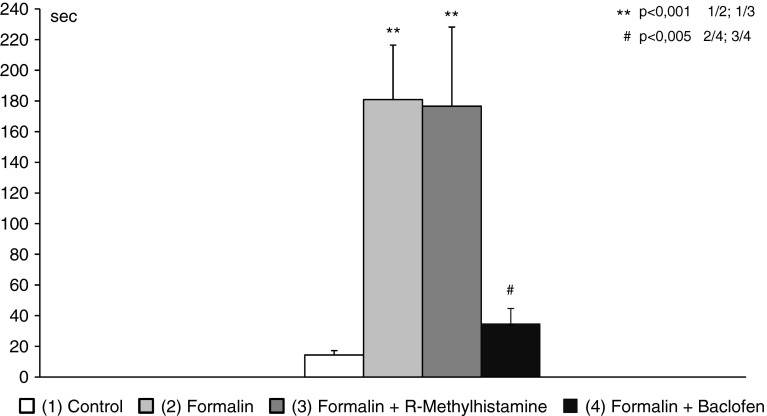
Antinociception effects measured by time of rubbing, after locally applied R- alpha-methylhistamine (12.5 μg) and baclofen (50 μg), as assessed in the orofacial formalin test in rats (x ± SEM; *n* = 8). *Open square* Control, *Light grey box* Formalin, *dark grey box* Formalin + R-alpha-methylhistamine, *black box* Formalin + baclofen

Control animals (saline + saline) spent 14.0 ± 2.78 s on rubbing their snout. Rats in the formalin group rubbed their snout for 180.8 ± 35.5 s, similarly to rats pretreated with R-methylhistamine (176.6 ± 51.4 s) before formalin injection. In contrast, baclofen attenuated nociceptive behavior (34.8 ± 9.75 s) induced by formalin (Fig. 2).

### Assessment of Biogenic Amine and Metabolite Content

**Fig. 3:**
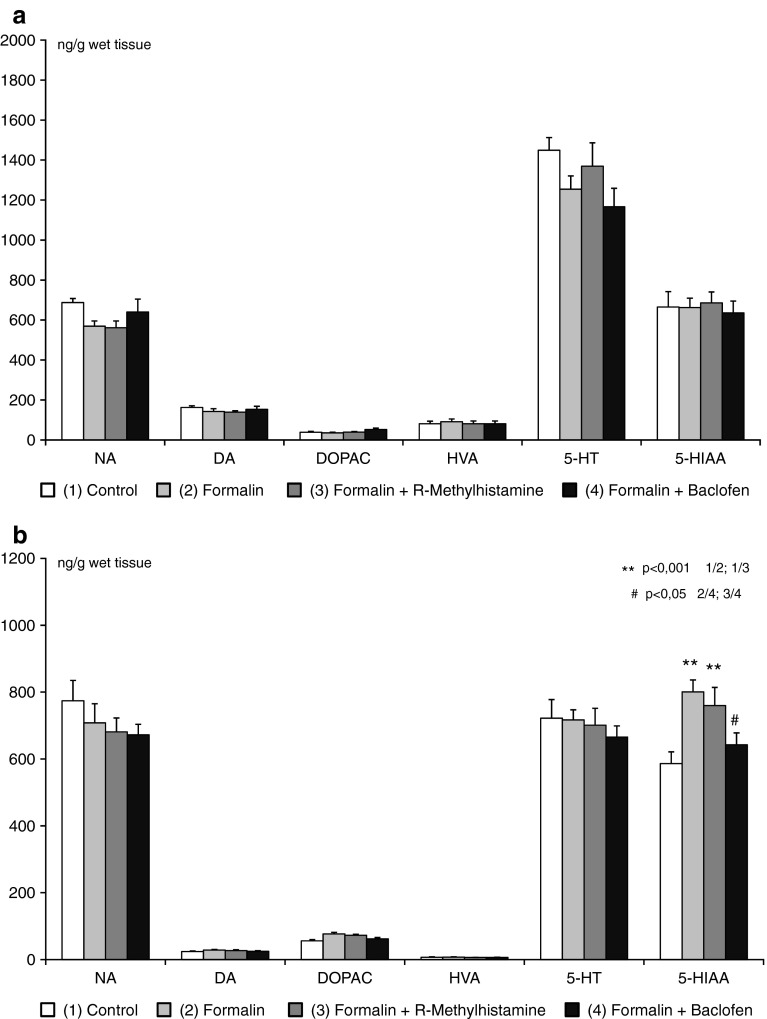

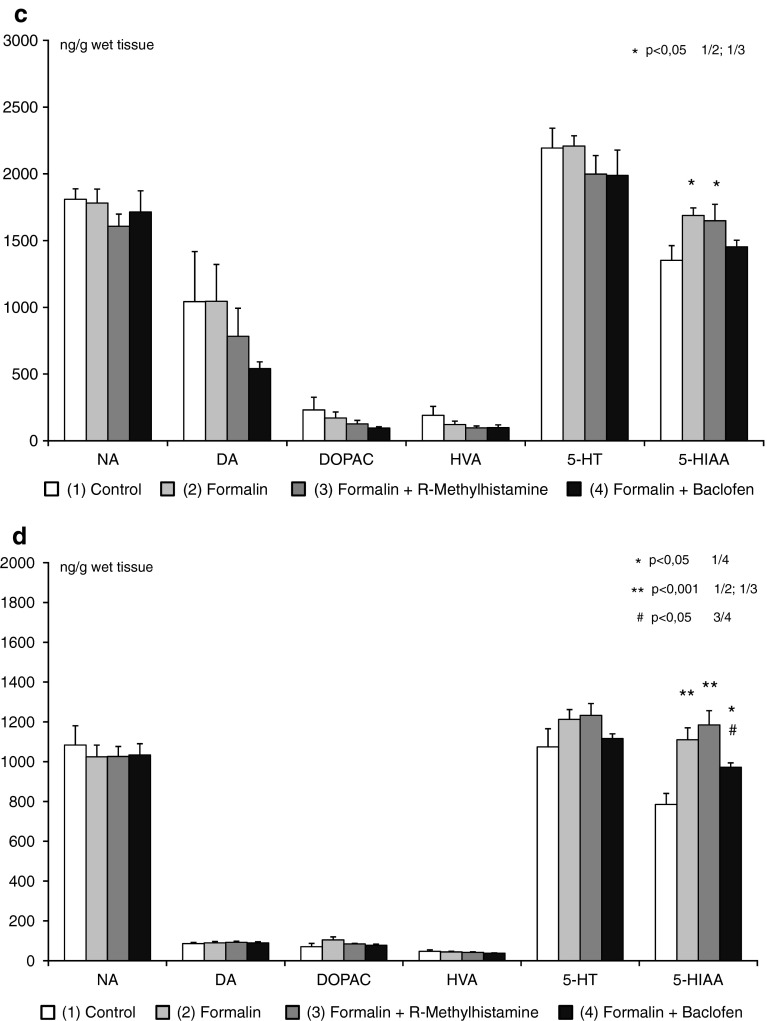
Monoamine and metabolite levels in the prefrontal cortex (**a**), hippocampus (**b**), thalamus (**c**), and brain stem (**d**) after locally applied R-alpha-methylhistamine (12.5 μg) and baclofen (50 μg), as assessed in the orofacial formalin test in rats (x ± SEM; *n* = 8). Legend as in Figure 1

Equally high levels of DA, DOPAC, HVA, 5-HT, and 5-HIAA in the prefrontal cortex were observed between all groups of rats (control, formalin, formalin + R-alpha-methylhistamine, and formalin + baclofen animals). A borderline significance (*p* < 0,088) was observed in NA content between control and formalin group (Fig. 3a–d). Also in other tested brain structures, equally high levels of NA, DA, DOPAC, HVA, and 5-HT were noted. Conversely, in the hippocampus, thalamus and brain stem of rats treated with formalin and formalin + R-alpha-methylhistamine, 5-HIAA content was increased, and with baclofen pretreatment 5-HIAA content was unchanged from control (Fig. 3b–d).

## Discussion

The rat orofacial formalin test is a useful pre-clinical model of inflammatory trigeminal pain for evaluating antinociceptive activity of analgesics and their combinations. Injection of formalin in the rat wiskerpad induces jumping behavior and stereotyped response (rubbing), consisting of two distinct phases: a first “phasic” phase and a second “tonic” phase (Raboisson and Dallel 2004). In this work, we tested R-alpha-methylhistamine a histamine H_3_ receptor agonist and baclofen a GABA_B_ receptor agonist, each of which was given locally into the rat right upper lip. We have shown that baclofen reduced nociception both in the first (jumping) and in the second phase (rubbing), whereas R-alpha-methylhistamine induced antinociception in the first phase only. Our results (with baclofen) are in line with Whitehead et al. (2012) who found that GABA_B_ agonists like isovaline, baclofen, and GABA (a CNS-impermeant, unselective GABA_B_ agonist) attenuated allodynia induced by prostaglandin E_2_ injection into the mouse hindpaw and tested with von Frey filaments. They also demonstrated that immunohistochemical staining of cutaneous layers of the analgesic test site demonstrated co-localization of GABA_B1_ and GABA_B2_ receptor subunits on fine nerve endings and keratinocytes, which may account for antinociceptive effects. The only difference versus our study is that they administered agents systemically, not peripherally, so that the analgesic effect was also dependent on modification of nociceptive transmission in the trigeminal root ganglion neurons as well as in upper brain structures (Takeda et al. 2004). It is noteworthy that there are also some clinical observations on this topic. For example, Sanders et al. (2009) showed that intrathecal baclofen decreases acute and chronic postoperative pain after total knee arthroplasty. Patients from the baclofen group used less morphine than the control group.

We also found that R-alpha-methylhistamine-induced antinociception in the first phase of orofacial formalin test by decreasing number of jumps without an effect on the “inflammatory” test phase. Conversely, Fernández-Dueñas et al. (2010b) found that the systemic administration of fentanyl (0.005–0.1 mg/kg) plus a fixed dose of R-alpha-methylhistamine (0.5 mg/kg) induced a supra additive effect on the inhibition of the thermal hyperalgesia and substance P accumulation in the hind-paw skin of inflamed mice. Others investigated the effects of systemically (s.c.) and intrathecally (i.t.) administered immepip (a histamine H_3_ receptor agonist) in rats and mice. They showed that immepip produced robust antinociception in rats on a mechanical (tail pinch) test but did not alter nociceptive responses on a thermal (tail flick) test. In contrast, this treatment in mice did not change either mechanically or thermally-evoked nociceptive responses (Cannon et al. 2003). In a more recent rat study, systemic (s.c.) administration of the H_3_ agonist immepip produced dose-dependent reductions (up to 70 %) in both phases of formalin-induced flinching. These effects were mimicked by i.t. immepip and blocked by systemic and i.t. thioperamide. Although s.c. immepip reduced formalin-induced flinching, this treatment had no effect on formalin-induced vocalizations in rats (Cannon et al. 2007). These results, in apparent contradiction to ours findings, could be the outcome of systemic versus local drug administration. One must be cognizant that histamine H_3_ receptors are widely distributed within the brain, the spinal cord and on specific types of primary sensory neurons. Our “manipulation” was restricted solely to H_3_ receptors located on certain Aβ fibers, keratinocytes, Merkel cells and peptidergic Aδ fibers terminating on deep dermal blood vessels in the skin (Hough and Rice 2011).

In our work, we also looked into brain monoamine metabolism. Immediately after the behavioral testing ended, brain specimens were taken for eventual HPLC analysis. We found that in the hippocampus, thalamus and brain stem of rats treated with formalin and formalin + R-alpha-methylhistamine, there was an increase in 5-HIAA levels, and baclofen pretreatment prevented a change in 5-HIAA levels. From these studies, we learn that persistent pain increased 5-HT metabolism in brain structures that are involved in pain perception. Based on the finding that baclofen relieved nociceptive effects and effectively maintained 5-HT levels, we hypothesize that 5-HT brain metabolism may be a simple biochemical indicator for the painful stimuli in rats. Our data are in accord with Burke et al. (2010) who found that 2 h after intra-plantar formalin administration in rats an increase in 5-HT and 5-HIAA concentration in the prefrontal cortex, hippocampus, thalamus; and 5-HIAA in amygdaloid cortex and cerebellum was observed. A similar “constellation” of marked effects upon 5-HIAA as opposed to 5-HT was found by Cox et al. (2011) who observed an increase in 5-HIAA, but no increase in 5-HT in the hypothalamus of rats exposed to a random pattern of mild stressors twice daily for 10 days (a model of chronic unpredictable stress). This resulted in an increased ratio of 5-HIAA/5-HT, suggesting increased turnover of 5-HT which is reflective of increased serotoninergic activity. An increase in 5-HT turnover in the hypothalamus was previously observed in response to chronic social stress (Blanchard et al. 1991).

The search for new peripherally restricted analgesics is desirable to avoid central nervous system side effects of opioids and overall toxicity of nonsteroidal anti-inflammatory drugs. Our work showed that GABA_B_ receptors represent peripheral targets for analgesia; locally administered baclofen may be a useful approach in treating inflammatory trigeminal pain.

## References

[CR1] Ataka T, Kumamoto E, Shimoji K, Yoshimura M (2000). Baclofen inhibits more effectively C-afferent than Adelta-afferent glutamatergic transmission in substantia gelatinosa neurons of adult rat spinal cord slices. Pain.

[CR2] Blanchard DC, Cholvanich P, Blanchard RJ, Clow DW, Hammer RP, Rowlett JK, Bardo MT (1991). Serotonin, but not dopamine, metabolites are increased in selected brain regions of subordinate male rats in a colony environment. Brain Res.

[CR3] Brown RE, Stevens DR, Haas HL (2001). The physiology of brain histamine. Prog Neurobiol.

[CR4] Burke NN, Hayes E, Calpin P, Kerr DM, Moriarty O, Finn DP, Roche M (2010). Enhanced nociceptive responding in two rat models of depression is associated with alterations in monoamine levels in discrete brain regions. Neuroscience.

[CR5] Cannon KE, Nalwalk JW, Stadel R, Ge P, Lawson D, Silos-Santiago I, Hough LB (2003). Activation of spinal histamine H_3_ receptors inhibits mechanical nociception. Eur J Pharmacol.

[CR50] Cannon KE, Leurs R, Hough LB (2007). Activation of peripheral and spinal histamine H_3_ receptors inhibits formalin-induced inflammation and nociception, respectively. Pramacol Biochem Behav.

[CR6] Cox BM, Alsawah F, McNeill PC, Galloway MP, Perrine SA (2011). Neurochemical, hormonal, and behavioral effects of chronic unpredictable stress in the rat. Behav Brain Res.

[CR7] Fernández-Dueñas V, Ciruela F, Gandía J, Sánchez S, Planas E, Poveda R (2010). Histamine H_3_ receptor activation potentiates peripheral opioid-mediated antinociception: substance P role in peripheral inflammation in mice. Eur J Pharmacol.

[CR8] Fernández-Dueñas V, Ciruela F, Gandía J, Sánchez S, Planas E, Poveda R (2010). Histamine H_3_ receptor activation potentiates peripheral opioid-mediated antinociception: substance P role in peripheral inflammation in mice. Eur J Pharmacol.

[CR9] Flik G, Dremencov E, Cremers TI, Folgering JH, Westerink BH (2011). The role of cortical and hypothalamic histamine-3 receptors in the modulation of central histamine neurotransmission: an in vivo electrophysiology and microdialysis study. Eur J Neurosci.

[CR10] Franek M, Vaculín S, Rokyta R (2004). GABA(B) receptor agonist baclofen has non-specific antinociceptive effect in the model of peripheral neuropathy in the rat. Physiol Res.

[CR11] Hasanein P (2010). Histamine H_3_ receptor modulates nociception in a rat model of cholestasis. Pharmacol Biochem Behav.

[CR12] Hough LB, Rice FL (2011). H3 receptors and pain modulation: peripheral, spinal, and brain interactions. J Pharmacol Exp Ther.

[CR13] Korossy-Mruk E, Kuter K, Nowak P, Szkilnik R, Rykaczewska-Czerwinska M, Kostrzewa RM, Brus R (2013). Neonatal DSP-4 treatment modifies antinociceptive effects of the CB_1_ receptor agonist methanandamide in adult rats. Neurotox Res.

[CR14] Koyrakh L, Lujan R, Colon J, Karschin C, Kurachi Y, Karschin A, Wickman K (2005). Molecular and cellular diversity of neuronal G-protein-gated potassium channels. J Neurosci.

[CR15] Lao L, Marvizón JC (2005). GABA_A_ receptor facilitation of neurokinin release from primary afferent terminals in the rat spinal cord. Neuroscience.

[CR16] Mapelli L, Rossi P, Nieus T, D’Angelo E (2009). Tonic activation of GABA_B_ receptors reduces release probability at inhibitory connections in the cerebellar glomerulus. J Neurophysiol.

[CR17] Nowak P, Szczerbak G, Biedka I, Drosik M, Kostrzewa RM, Brus R (2006). Effect of ketanserin and amphetamine on nigrostriatal neurotransmission and reactive oxygen species in Parkinsonian rats. In vivo microdialysis study. J Physiol Pharmacol.

[CR18] Park MK, Lee JH, Yang GY, Won KA, Kim MJ, Park YY, Bae YC, Ahn DK (2011). Peripheral administration of NR2 antagonists attenuates orofacial formalin-induced nociceptive behavior in rats. Prog Neuropsychopharmacol Biol Psychiatry.

[CR19] Pollard H, Moreau J, Arrang JM, Schwartz JC (1993). A detailed autoradiographic mapping of histamine H_3_ receptors in rat brain areas. Neuroscience.

[CR20] Potes CS, Neto FL, Castro-Lopes JM (2006). Inhibition of pain behavior by GABA_B_ receptors in the thalamic ventrobasal complex: effect on normal rats subjected to the formalin test of nociception. Brain Res.

[CR21] Raboisson P, Dallel R (2004). The orofacial formalin test. Neurosci Biobehav Rev.

[CR22] Rusina R, Barek S, Vaculin S, Azérad J, Rokyta R (2010). Cortical stimulation and tooth pulp evoked potentials in rats: a model of direct anti-nociception. Acta Neurobiol Exp (Wars).

[CR23] Sanders JC, Gerstein N, Torgeson E, Abram S (2009). Intrathecal baclofen for postoperative analgesia after total knee arthroplasty. J Clin Anesth.

[CR24] Suh HW, Chung KM, Kim YH, Huh SO, Song DK (1999). Effects of histamine receptor antagonists injected intrathecally on antinociception induced by opioids administered intracerebroventricularly in the mouse. Neuropeptides.

[CR25] Takeda M, Tanimoto T, Ikeda M, Kadoi J, Matsumoto S (2004). Activaton of GABA_B_ receptor inhibits the excitability of rat small diameter trigeminal root ganglion neurons. Neuroscience.

[CR26] Whitehead RA, Puil E, Ries CR, Schwarz SK, Wall RA, Cooke JE, Putrenko I, Sallam NA, MacLeod BA (2012). GABA_B_ receptor-mediated selective peripheral analgesia by the non-proteinogenic amino acid, isovaline. Neuroscience.

